# Analysis of glutathione levels in the brain tissue samples from HIV-1-positive individuals and subject with Alzheimer's disease and its implication in the pathophysiology of the disease process

**DOI:** 10.1016/j.bbacli.2016.05.006

**Published:** 2016-05-29

**Authors:** Tommy Saing, Minette Lagman, Jeffery Castrillon, Eutiquio Gutierrez, Frederick T. Guilford, Vishwanath Venketaraman

**Affiliations:** a Graduate College of Biomedical Sciences, Western University of Health Sciences, Pomona, CA 91766, USA; b Department of Basic Medical Sciences, College of Osteopathic Medicine of the Pacific, Western University of Health Sciences, Pomona, CA 91766-1854, USA; c Your Energy System, Palo Alto, CA, USA

**Keywords:** HIV, Glutathione, Oxidative stress, Opportunistic infections

## Abstract

HIV-1 positive individuals are at high risk for susceptibility to both pulmonary tuberculosis (TB) and extra-pulmonary TB, including TB meningitis (TBM) which is an extreme form of TB. The goals of this study are to determine the mechanisms responsible for compromised levels of glutathione (GSH) in the brain tissue samples derived from HIV-1-infected individuals and individuals with Alzheimer's disease (AD), investigate the possible underlying mechanisms responsible for GSH deficiency in these pathological conditions, and establish a link between GSH levels and pathophysiology of the disease processes. We demonstrated in the autopsied human brain tissues that the levels of total and reduced forms of GSH were significantly compromised in HIV-1 infected individuals compared to in healthy subjects and individuals with AD. Brain tissue samples derived from HIV-1-positive individuals had substantially higher levels of free radicals than that derived from healthy and AD individuals. Enzymes that are responsible for the *de novo* synthesis of GSH such as γ-glutamate cysteine-ligase catalytic subunit (GCLC-rate limiting step enzyme) and glutathione synthetase (GSS-enzyme involved in the second step reaction) were significantly decreased in the brain tissue samples derived from HIV-1-positive individuals with low CD4 + T-cells (< 200 cells/mm^3^) compared to healthy and AD individuals. Levels of glutathione reductase (GSR) were also decreased in the brain tissue samples derived from HIV-1 infected individuals. Overall, our findings demonstrate causes for GSH deficiency in the brain tissue from HIV-1 infected individuals explaining the possible reasons for increased susceptibility to the most severe form of extra-pulmonary TB, TBM.

## Highlights

• Total and reduced forms of GSH were significantly compromised in the brain tissues derived from HIV-1 infected individuals.
• Brain tissue samples derived from HIV-1-positive individuals had substantially higher levels of free radicals.
• GSH de novo synthesis enzymes were significantly decreased in HIV-1-positive individuals with low CD4 + T-cells.
• Levels of GSR were also decreased in the brain tissue samples derived from HIV-1 infected individuals.
• Overall, our findings demonstrate causes for GSH deficiency in the brain tissue from HIV-1 infected individuals.

## 1 Introduction

As of 2010, an estimated 34 million people are living with HIV-1 infection worldwide, with an additional 2.7 million people newly infected each year. Of those 34 million living with HIV-1, 22.9 million live in sub-Saharan Africa, a region where *Mycobacterium tuberculosis* is endemic [1], [2], [3], [4]. One of the hallmarks of acquired immunodeficiency syndrome (AIDS) brought on by HIV-1 infection is increased susceptibility to opportunistic infections [1], [2], [3], [4]. Tuberculosis (TB) is the most prevalent infectious disease in the world. It is also believed that in developing countries, as many as 40 to 80% of individuals with AIDS are at risk of developing TB. In recent years there has been a significant increase in the incidence of TB due to the emergence of multi-drug resistant strains of *M. tb* and due to increased numbers of highly susceptible immuno-compromised individuals arising from the AIDS pandemic [1], [2], [3], [4], [5], [6], [7], [8], [9], [10]. Extra-pulmonary TB has become more common since the advent of HIV-1 infection and is seen in more than 50% of patients with concurrent AIDS and TB [5], [6], [7], [8], [9], [10]. The risk of extrapulmonary TB and mycobacteremia increases with advancing immunosuppression [5], [6], [7], [8], [9], [10]. TBM is the most common and severe form of extra-pulmonary TB and is associated with significant morbidity and mortality [5], [10].

GSH is critical for a number of cellular functions including protein synthesis, apoptosis, and transmembrane transport, enzyme catalysis [11], [12]. Establishing proper levels of GSH is also critical for the maintenance and regulation of the thiol-redox state of the cell [13], [14], [17], [18]. GSH is produced *de novo* from a tripeptide composed of the amino acids glutamine, cysteine, and glycine. GSH exists intracellularly in two forms: oxidized form (GSSG) and the reduced form (rGSH). Formation of rGSH occurs in two-steps synthesis involving two enzymes, glutamate-cysteine ligase (GCL) and glutathione synthase (GSS). GCL catalysis the first step (also the rate limiting step) reaction involved in the synthesis of GSH and is composed of a catalytic (GCLC) and a modifier (GCLM) subunit [14], [22]. Alternatively, GSSG can be converted back to GSH in the presence of the enzyme glutathione reductase (GSR) using cofactor NADPH [14].

We observed *in vitro* that the virulent *M. tb* strain is sensitive to GSH at physiological concentrations (5 mM) [21]. We also found that enhancing the levels of GSH in human macrophages by treatment with either *N*-acetyl cysteine (NAC) or with rGSH formulated in liposomes (L-GSH) significantly reduced the intracellular growth of *M. tb* [11]. Thus, GSH has direct antimycobacterial activity and also functions as an effector molecule in innate defense against *M. tb* infection [11], [15], [16], [19], [20], [21]. We have reported that GSH, in combination with cytokines such as IL-2 and IL-12, enhances the activity of natural killer (NK) cells to inhibit *M. tb* growth inside human monocytes [33]. We also showed that GSH activates T cell functions to control *M. tb* infection inside human monocytes [16]. Finally, we demonstrated that the total and reduced forms of GSH were significantly compromised in macrophages, NK cells, and T cells isolated from the peripheral blood of HIV-1-infected individuals [15], [19], [33], [16]. Decreased levels of GSH in individuals with HIV-1 infection were accompanied by diminished levels of enzymes, such as GCLC and GSS in the red blood cells [34]. We also established that compromised levels of GSH in immune cells derived from the peripheral blood of individuals with HIV-1 infection led to increased survival of *M. tb* inside macrophages [15], [16], [19]. Augmenting the levels of GSH in macrophages derived from individuals with HIV infection resulted in improved control of *M. tb* infection [15], [19]. Furthermore, cytokines that are responsible for controlling intracellular infections such as TNF-α, IL-1β, IL-2, IFN-γ, and IL-12 were found to be compromised, while IL-10, an immunosuppressive cytokine was elevated in the plasma samples of HIV-1-infected individuals [20], [35]. We have successfully demonstrated that supplementing individuals with HIV-1 infection (CD4 T cell counts between 200–900/mm^3^) for 13 weeks orally with L-GSH (supplied by Your Energy Systems) significantly increased the levels of cytokines, such as IL-2, IL-12 and IFN-γ, which are important for mediating effective immune responses against intracellular infections [20]. L-GSH supplementation in HIV-1-infected individuals also resulted in a substantial decrease in the levels of free radicals and immunosuppressive cytokines such as IL-10 and TGF-β, relative to those in a placebo-controlled cohort [20]. We observed that the oxidative stress in HIV-1 infection is due to a systemic increase in IL-6 production triggered by the virus and this is further enhanced by diminished levels of GSH. Elevations of TGF-β are associated with a number of factors that may perpetuate immunosuppression in HIV-1 infection [15], [35]. Oral L-GSH ingestion was able to decrease TGF-β and increase GSH levels in the plasma of HIV-1-infected individuals [20]. Our study [20] showed that IL-10, an immunosuppressive cytokine remains elevated in HIV-1-infected individuals on successful HAART therapy. L-GSH can lower the level of IL-10 after 13 weeks of oral ingestion [20]. Taken together, we showed that L-GSH supplementation to HIV-1-infected individuals improved their T-helper-1 (TH1) response, which is associated with enhanced defense against intracellular infections. Our findings imply that oxidative stress and redox imbalance that occur in HIV-1 infection often leads to inappropriate immune responses and maintaining redox homeostasis result in host-protective immune responses against intracellular infections [20].

The methylation and transsulfuration pathways are intimately linked and have been implicated in the progression of neurologic damage and immune cell depletion caused by HIV-1 infection [26]. Metabolites related to methylation and transsulfuration pathways include: S-adenosylmethionine (SAMe), homocysteine, cysteine, cysteinyl-glycine, and GSH [26]. It has been shown in HIV-1-infected patients that the levels of GSH, SAMe and cysteinyl-glycine, but not homocysteine or cysteine, were significantly reduced in the cerebrospinal fluid (CSF) [26]. Administration of SAMe-butanedisulphonate, 800 mg/d intravenously for 14 days, was associated with significant increases in CSF SAMe and GSH [26]. However, conflicting results were obtained when the GSH levels were determined in the brain tissue lysates from HIV-1-transgenic rats [32].

In this study, we examined the levels of GSH and enzymes involved in the synthesis of GSH in autopsied human brain tissues of HIV-1-infected individuals. Disturbances in GSH homeostasis are implicated in the etiology and/or progression of a number of neurodegenerative diseases including AD [36], [37], [38]. GSH deficiency or a decrease in the GSH/GSSG ratio manifests itself largely through an increased susceptibility to oxidative stress, and the resulting damage is thought to be involved in the pathogenesis of AD [36], [37], [38]. Since GSH deficiency plays an important role during the onset and progression of AD, we also wanted to examine the levels of GSH and causes for GSH deficiency in the brain tissues derived from individuals with AD, a neurodegenerative condition with increased oxidative stress. Our results suggest an important mechanism that is responsible for decreased levels of GSH in the brain tissue samples derived from individuals with HIV-1 infection which could represent a risk factor for the onset of TBM.

## 2 Materials and methods

### 2.1 Subjects

All assays in this study were conducted in human brain tissue samples (frontal cortex region) obtained from three different groups such as 1) non-demented healthy group (control) 2) AD patients 3) and non-demented HIV-1 infected patients (with low and high CD4 T cell counts). We did not use samples from individuals with co-morbidity such as HIV + AD. AD and non-demented control cases were obtained from the University of California-Irvine – ADRC Brain Tissue Repository. AD cases were age-matched with normal healthy control. The average ages between the AD and normal control subjects were 86 years old. Inclusion criteria for AD group required that all subjects had AD with severe plaques and tangles. The exclusion criteria ruled out any meningitis, trauma to the head, and any viral and bacterial infections in brain tissues.

The HIV-1 infected cases were obtained from the National NeuroAIDS Tissue Consortium (NNTC) at U.C. San Diego. The HIV-1 subjects were divided into two groups: HIV-1-positive with CD4 + < 200 cells/mm^3^ and CD4 + > 200 cells/mm^3^ and were labeled as low and high CD4 +, respectively. The average ages for the low CD4 + group were 42 years, and 52 years for high CD4 + group.

### 2.2 Sample preparation for bioassay measures

Upon receipt, brain tissues (frontal cortex) from AD, non-demented healthy controls, and non-demented HIV-1 were stored in − 80 °C. On the day of sample preparation, brain tissues from the three experimental groups were removed from − 80 °C and thawed in PBS lysis buffer (pH 7.4) containing 320 mM sucrose, 1% of 1.0 M Tris-HCl (pH = 8.8), 0.098 mM MgCl_2_, 0.076 mM EDTA, phosphatase inhibitor cocktail (Sigma Aldrich, St. Louis, MO). The brain tissues were homogenized by 10 quick pulses using the hand held homogenizer. The homogenates were centrifuged at 14,000 ×* g* for 10 min to remove cellular debris. The supernatants were separated in order to determine the total protein concentration using the BCA method (Pierce, Rockford, IL). Additional extraction was performed with RIPA buffer (50 mM Tris-HCl pH 7.4, 150 mM NaCl, 1% Triton x-100, 1% Sodium deoxycholate, 0.1% SDS, 1 mM EDTA, Protease Inhibitors [Roche]). Brain tissue lysates were then stored in − 80 °C until use.

### 2.3 Measurement of GSH

Total, oxidized and reduced forms of GSH were measured in the brain lysates from normal healthy controls, AD, and HIV-1 groups. A GSH detection kit from Arbor Assays (K006-H1) was used to measure concentrations of total, oxidized and reduced forms of GSH. All tissue lysates were deproteinized with 5% Sulfosalicylic acid (SSA) to help remove any protein thiols present, and slow oxidation of rGSH. The SSA concentration levels were brought down to 1% by dilution of assay buffer before analysis. Samples were then assayed for total, free and oxidized GSH following the manufacturer's protocol. Results were corrected for protein levels and were reported in μmoles GSH/g protein.

### 2.4 Measurement of oxidative stress or reactive oxygen species (ROS)

ROS production was determined by measurement of malondialdehyde or MDA (end product of lipid peroxidation-an indirect measure of ROS production) in brain lysates. MDA is a byproduct of lipid peroxidation. Once MDA forms an adduct with thiobarbituric acid (TBARS) at 90 °C − 100 °C, a color change occurs which can be measured colorimetrically at 530–540 nm. Baseline levels of MDA were measured in brain lysates. MDA levels were measured using a TBARS Assay Kit (Cayman Chemical, 10009055). The assay procedure included with the kit was followed to obtain MDA sample concentrations. Results were corrected for protein levels and were reported in μmoles MDA/g protein.

### 2.5 Western blot analysis of GSH synthesis enzymes in brain lysates

Total protein concentration in the brain lysates was measured using the Coomassie blue colorimetric assay. A total of 35 μg of total brain lysate proteins per sample were separated by denaturing polyacrylamide electrophoresis with 12% polyacrylamide. The membranes were blocked for one hour at room temperature with Tris buffered saline containing tween-20 (TBST) and 5% nonfat dry milk, with mild shaking. The membranes were then incubated overnight with primary antibody diluted in 5% nonfat dry milk blocking buffer at 4 °C with mild shaking. Primary antibodies were GCLC (1:1000; Abcam), GSS (1:1000; Abcam), GSR (1:250; Abcam) and Glyceraldehyde phosphate dehydrogenase (GAPDH 1:1000; Abcam). Following incubation with primary antibody, the membranes were washed five times with TBST for 5 min at room temperature. The membranes were then incubated at room temperature for one hour with horseradish peroxidase-conjugated goat anti-rabbit (Millipore Cat# 12-348) or goat anti-mouse (Millipore Cat # 12-349) secondary antibody diluted with TBST followed by another set of washing with TBST. Chemiluminescent substrate (Thermo Fisher Scientific) was added onto the membrane followed by exposure to the X-ray film. Following this, the X-ray film was developed in the dark room. Resulting immunoblots were captured by Versadoc and densitometrically analyzed by Image J.

### 2.6 Measurement of cytokines – IL-1 and TNF-α

Cytokine levels were measured using Enzyme-linked Immunosorbent Assay (ELISA). The cytokines that were measured in the brain tissue lysates isolated from the subjects IL-1β and TNF-α (eBioscience ELISA Ready-Set-Go: IL-1β cat # 88-7010, TNF-α cat # 88-7346). The cytokine levels were measured following the manufacturer's protocol.

### 2.7 Statistical analysis

Statistical data analysis was performed using Graph Pad Prism Software version 6. Levels of total GSH, rGSH, GSH synthesis enzymes, MDA and cytokines were compared between non-demented healthy controls, AD, and non-demented HIV-1 with low and high CD4 + T cell counts group using the unpaired *t*-test with Welch correction. Reported values are in means, p < 0.05 was considered significant.

## 3 Results

### 3.1 Assay of GSH in brain lysates

**Fig. 1:**
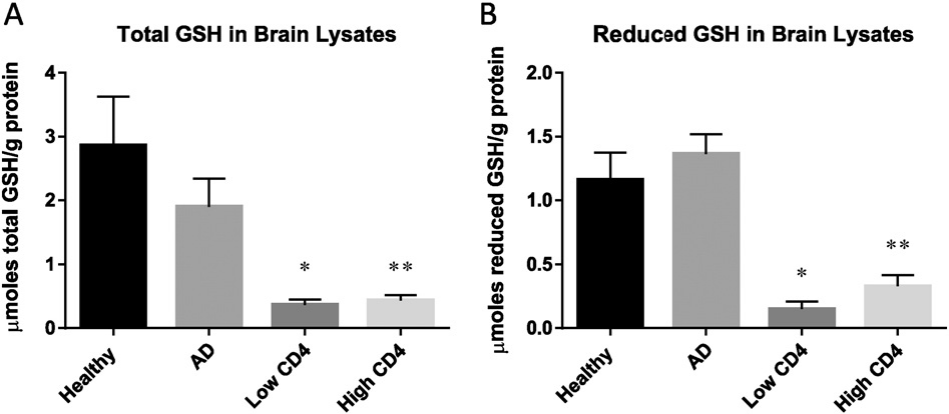
Assay of total GSH and rGSH in brain lysates from healthy, AD, and HIV-1 positive subjects. Frontal cortex specimen was homogenized and extracted with PBS buffer. GSH assay was performed using a colorimetric assay kit from Arbor Assays. Data represent means ± SE from 5 healthy individuals, 5 individuals with AD and 10 HIV-1-positive subjects, *p < 0.05, **p < 0.05. HIV-1-positive subjects were sub-divided by CD4 + T-cell counts: low CD4 + T-cell < 200 cells/mm^3^ and high CD4 + T-cell > 200 cells/mm^3^. Panel A: total GSH were significantly lower from HIV-1-positive subjects with low CD4 + T-cells and high CD4 + T-cells compared to healthy subjects. Data represents means ± SE from 5 healthy subjects, 5 AD, and 5 HIV-1-positive individuals *p < 0.05, **p < 0.05. No statistical significance was shown between healthy and AD patients. Panel B: there was a significant decrease in the levels of rGSH in HIV-1-positive subjects with low CD4 + T-cells and high CD4 + T-cells compared to healthy subjects. Data represents means ± SE from 5 healthy subjects, 5 AD, and 5 HIV-1-positive individuals *p < 0.05, **p < 0.05.

Our analysis of the levels of total and reduced forms of GSH in the frontal cortex of HIV-1-infected individuals showed a significant decrease compared to healthy and AD individuals (Fig. 1). Total GSH levels in AD subjects were lower than the healthy controls, however the decrease was not statistically significant (Fig. 1A). Further analysis of HIV-1-positive individuals showed that subjects with low and high levels of CD4 + T-cells had significantly diminished levels of total and reduced forms of GSH compared to healthy individuals (Fig. 1A,B). There was a significant and 3-fold decrease in the levels of rGSH in the brain tissues from HIV-1 subjects compared to AD (Fig. 1B).

### 3.2 Quantifying the levels of GSH enzymes GCLC, GSS, GSR

**Fig. 2:**
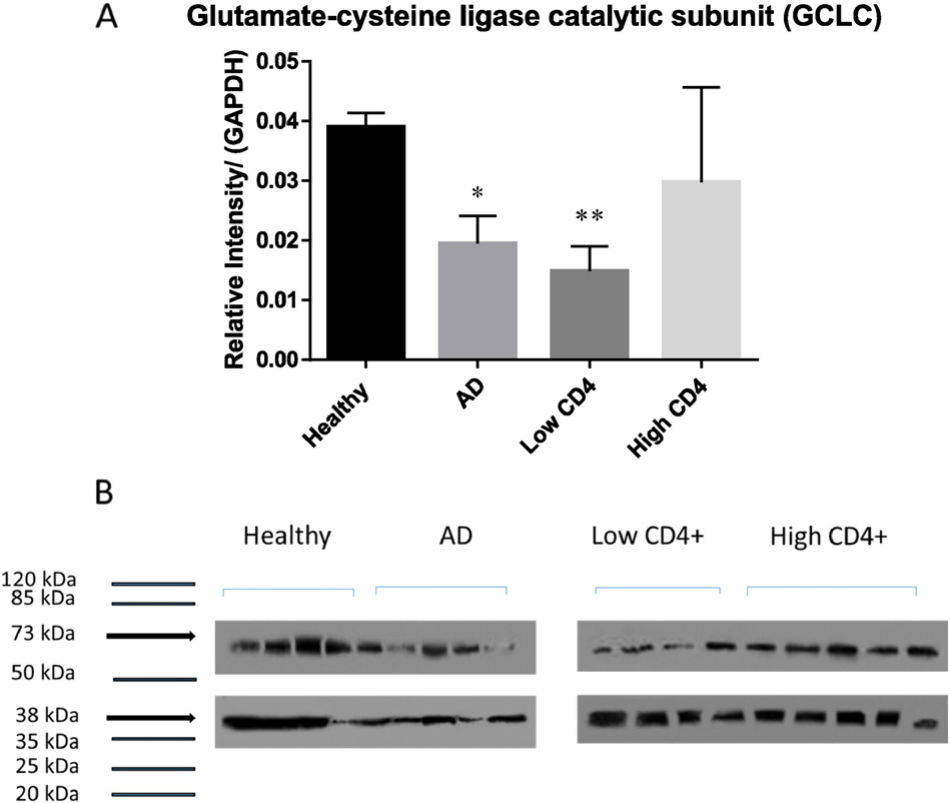
Western blot analysis of a GSH *de novo* synthesis enzyme, GCLC, in brain tissue from healthy subjects, AD and HIV-1-positive subjects. Data represent means ± SE,*p < 0.05. Panels A and B: Western blot results were corrected for GAPDH and reported as relative intensity compared to GAPDH. Levels of GCLC were significantly lower from HIV-1-positive subjects compared to healthy and AD subjects (B; n = 5 healthy, n = 4 AD, n = 5 HIV-1-positive). HIV-1-positive subjects with low CD4 + T-cells were observed to have significant decrease in the levels of GCLC compared to healthy subjects. No significant difference was observed with HIV-1-positive subjects with high CD4 + T-cells compared to healthy subjects.

**Fig. 3:**
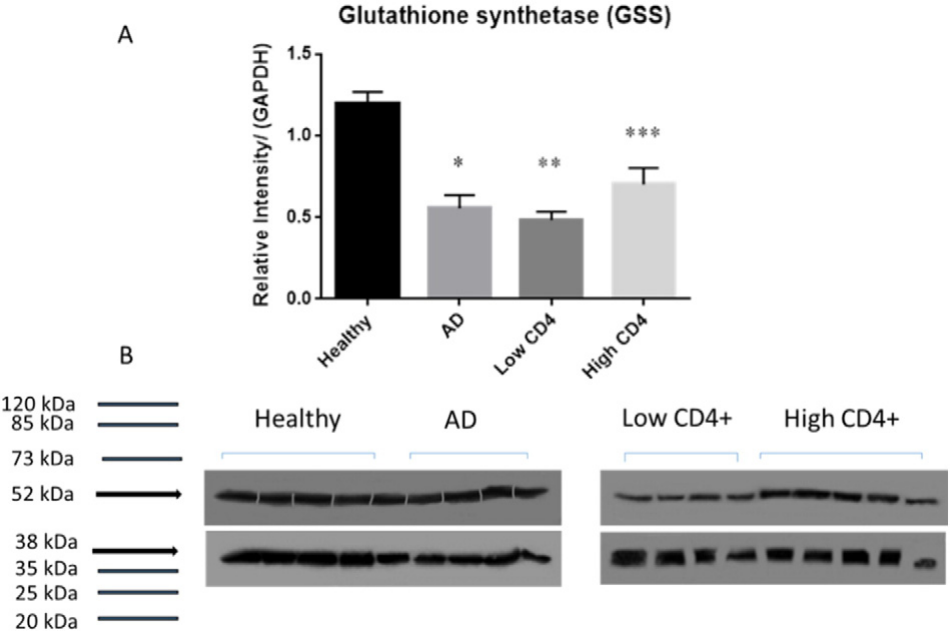
Western blot analysis of GSS, a GSH *de novo* synthesis enzyme in brain tissues derived from healthy, AD and HIV-1-positive subjects. Data represent means ± SE,*p < 0.05. Panels A and B: Western blot results were corrected for GAPDH and reported as relative intensity compared to GAPDH. Compared to healthy subjects, we observed levels of GSS to be significantly lowered from HIV-1-positive subjects with low CD4 + T-cells and high CD4 + T-cells and AD subjects (C; n = 5 healthy, n = 4 AD, n = 5 HIV-1-positive).

**Fig. 4:**
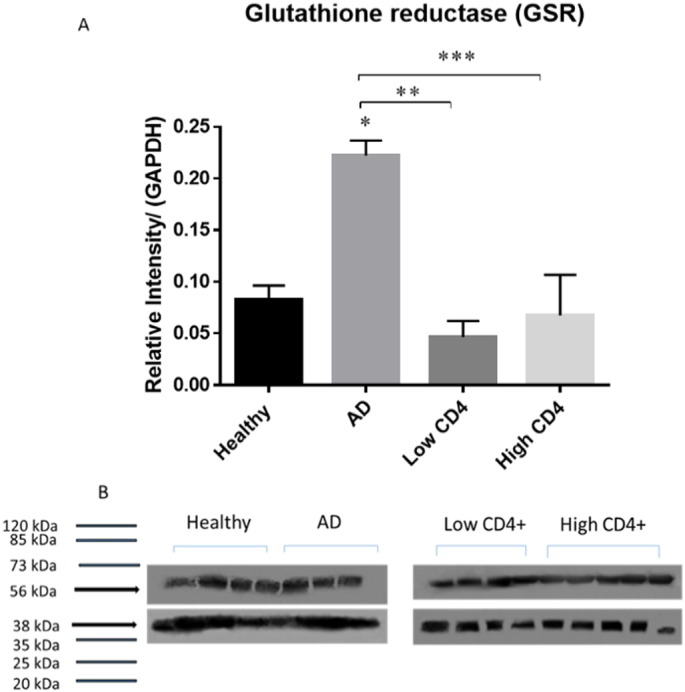
Western blot analysis of a recycling enzyme, GSR, in brain lysates from healthy, AD and HIV-1-positive subjects. Data represent means ± SE,*p < 0.05. Panels A and B: Western blot results were corrected for GAPDH and reported as relative intensity compared to GAPDH. We observed a significant increase in the levels of GSR in AD group compared to healthy group. We observed a significant decrease in the levels of GSR from HIV-1-positive subjects with low and high CD4 + T-cells compared to AD subjects (C; n = 5 healthy, n = 4 AD, n = 5 HIV-1-positive). *p < 0.05, **p < 0.05. ***p < 0.05.

We elucidated the causes for compromised levels of GSH, by demonstrating by Western blot analysis that the levels of GCLC (catalytic subunit of the enzyme responsible for catalyzing the fist-step reaction involved in the GSH synthesis) were significantly compromised in AD and HIV-1 positive individuals with low levels of CD4 + T-cells compared to the healthy group (Fig. 2A,B). Interestingly, the levels of GCLC were lower from HIV-1 positive subjects with low CD4 + T-cells compared to AD (Fig. 2A). Levels of GSS (enzyme involved in the second step of GSH synthesis) were also observed to be significantly lower in AD and HIV-1 positive individuals (both low and high CD4 + T-cell counts) compared to healthy individuals (Fig. 3A and B). In comparison to the healthy and HIV-1 groups, the levels of GSR (enzyme that converts GSSG back to rGSH *via* NADPH) were significantly higher in AD individuals (Fig. 4A and B). These results indicate that compromised levels of GSR may be an additional contributing factor for the decreased levels of GSH in the brain tissue of individuals with HIV-1 infection.

### 3.3 Measurement of free radicals in brain lysates

Levels of MDA in brain tissues of AD and HIV-1 groups were compared to healthy controls.

**Fig. 5:**
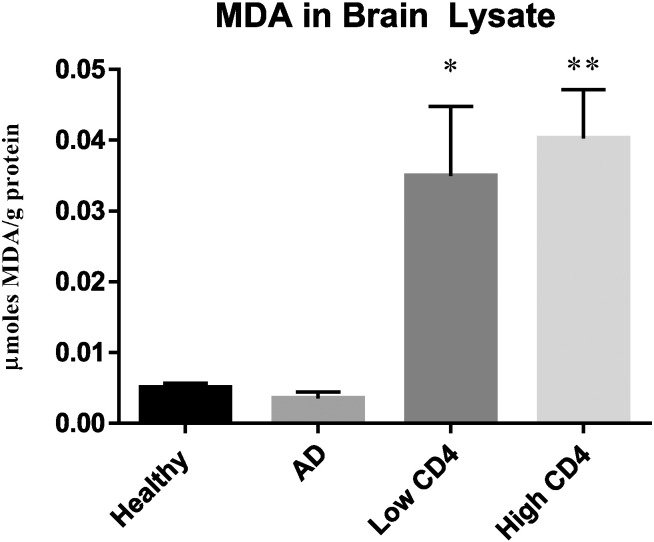
Assay of MDA in brain tissues from healthy, AD, and HIV-1-positive subjects. MDA assay was performed using a TBARS kit from Cayman Chemical. Data represent means ± SE from 5 healthy individuals, 5 AD subjects and 5 HIV-1-positive subjects *p < 0.05, **p < 0.05. There was a statistical significant increase in the levels of MDA in HIV-1-positive subjects with both low and high CD4 + T-cell counts compared to the healthy group.

Lipid peroxidation is a process that is involved in lipid degradation due to free radical formation. Free radicals are highly reactive and lead to oxidative damage in cells, including lipids and proteins. Free radicals attack polyunsaturated fatty acids and through the oxidative process, MDA, one of the final products is formed. Increased production of MDA is the result of an increase in free radicals found in the cells. MDA is a commonly used marker for measuring oxidative stress and in this study, we measured MDA levels in the brain tissues of healthy, AD, and HIV-1 individuals. Levels of MDA were comparable between healthy and AD individuals (Fig. 5). There was a significant increase in the levels of MDA in HIV-1 infected individuals with both low and high CD4 + T-cell subgroups compared to healthy and AD groups (Fig. 5).

### 3.4 Assay of pro-inflammatory cytokines IL-1β and TNF-α in brain tissues

**Fig. 6:**
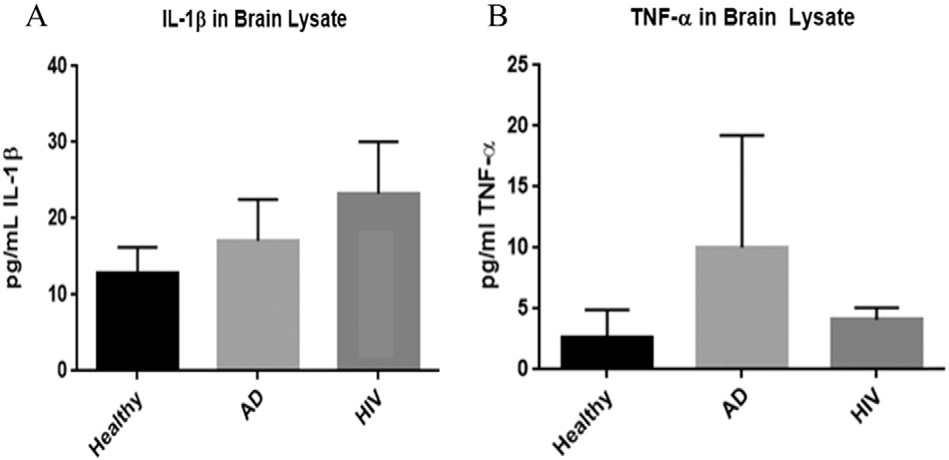
Assay of IL-1β (panel A) and TNF-α (panel B) in brain tissues from healthy, AD, and HIV-1-positive subjects. Data represent means ± SE from 5 healthy individuals, 5 AD subjects and 5 HIV-1-positive subjects *p < 0.05, **p < 0.05. Panels A and B: assay of IL-1β (panel A) and TNF-α (panel B) were performed using an ELISA Ready-Set-Go kit from eBioscience. Compared to healthy subjects, HIV-1-positive group was observed to have increased levels of IL-1β compared to healthy subjects. We observed a decrease level of TNF-α in HIV-1-positive subjects compared to healthy subjects.

We quantified the levels of pro-inflammatory cytokines such as IL-1β and TNF-α by performing a sandwich ELISA. There was an observable increase in the levels of the pro- inflammatory cytokine, IL-1β in the frontal cortex of HIV-1-positive individuals compared to healthy and AD brain tissues (Fig. 6A). Previously, our laboratory showed elevated levels of IL-1β in macrophages of HIV-1 infected individuals compared to healthy [15]. An important pro-inflammatory cytokine TNF-α, which is responsible for granuloma formation and controlling *M. tb* infection in macrophages were lower in HIV-1 infected individuals compared to healthy individuals and individuals with Alzheimer's disease (Fig. 6B).

## 4 Discussion

GSH is a tripeptide with important immunological functions. GSH deficiency is implicated in the etiology of human pathologies, including cancer, diseases of aging and neurodegenerative diseases [12]. Our laboratory has previously reported that GSH has direct antimycobacterial effects and immune-enhancing effects against *M. tb* [15], [21]. Furthermore, the levels of GSH are diminished in HIV-1 positive individuals and in individuals with type 2 diabetes [11], [15], [16], [20]. We recently demonstrated that supplementing HIV-1 positive individuals with GSH formulated in liposomes restored the levels of GSH and cytokines, and improved the immune responses against *M. tb* infection [15], [20].

In the current study, we demonstrate that the levels of total and reduced forms GSH are significantly compromised in the brain tissue samples derived from HIV-1 positive individuals (with low and high CD4 + T-cells) compared to healthy subjects and individuals with AD (Fig. 1A,B). These results indicate that GSH deficiency in the brain tissue can represent a risk factor for susceptibility to TBM, a severe and fatal form of extra-pulmonary TB [5].

There was a significant decrease in the levels of both GCLC and GSS enzymes in the brain tissue lysates of HIV-1 positive individuals with low CD4 + T cell counts compared to the healthy individuals (Fig. 2, Fig. 3). HIV-1 positive individuals with high CD4 + T cell counts had a significant decrease in the levels of GSS enzyme (but not GCLC) in their brain tissue lysates (Fig. 2, Fig. 3). There was no significant difference in the levels of GCLC and GSS enzymes in HIV-1 positive individuals between the low and high CD4 T-cell subgroups. In comparison to the healthy group, there was also a significant decrease in the levels of both GCLC and GSS enzymes in the brain tissue lysates from individuals with AD (Fig. 2, Fig. 3). As part of the *de novo* synthesis, these two enzymes (GCL and GSS) are critical for maintaining proper balance between both oxidative and antioxidant forms in the cells [13], [15], [19].

Furthermore, we also observed a significant increase in the levels of GSR in individuals with AD compared to the healthy group (Fig. 4A,B). Although there was a decrease in the levels of GSR in HIV-1 infected individuals with low and high CD4 + T-cells compared to the healthy group, this decrease was not significant (Fig. 4A,B). However, there was a statistically significant decrease in the levels of GSR between AD group and HIV-1 positive individuals with both low and high CD4 + T-cells (Fig. 4A,B). An increase in the levels of GSR in the brain tissue samples from individuals with AD indicates that there is a compensatory mechanism to restore the levels of GSH due to compromised levels of GCLC and GSS enzymes (Figs. 2A, 3A). This compensatory mechanism explains why there is no notable decrease in the levels of GSH in the AD group. Our observations demonstrating increased levels of GSR in the brain tissue lysates from AD group is consistent with the previously published findings in Parkinson diseases and other neurodegenerative diseases demonstrating increased expression of GSR as a compensatory mechanism to restore the levels of GSH that is being depleted due to chronic oxidative stress [30], [31].

We reported in our previous studies that individuals with HIV-1 infection have increased levels of TGF-β in their plasma and macrophage supernatants [15], [20]. TGF-β is known to block the production of GCLC which leads to decreased GSH synthesis [27]. HIV-1 transactivator protein (TAT) has been shown to decrease the amount of GSH through the modulation of GSH biosynthetic enzymes [28]. TAT also increases free radical production [28]. Therefore, marked increase in oxidative stress along with increased levels of TGF-β can diminish the production of enzymes that are involved in the synthesis of GSH. The master transcription factor nuclear factor (erythroid-derived 2)-like 2 (Nrf2) regulates the expression of antioxidant and phase II-metabolizing enzymes by activating the antioxidant response element (ARE) and thereby protects cells and tissues from oxidative stress [25]. The Nrf2 gene binding to the ARE results in the upregulation of GSH synthesis enzymes such as GCLC, GCLM, and GSR. New findings argue that HIV-1 related proteins downregulate Nrf2 expression and/or activity, which in turn impairs antioxidant defenses, thereby rendering the organs susceptible to oxidative stress and injury [29]. These studies support our current findings and elucidate the possible causes for decreased levels of GSH and diminished expressions of GCLC and GSS in the brain tissue samples derived from individuals with HIV-1 infection. Inability to induce substantial upregulation of GSR further explains disruption in the compensatory mechanism in the brain tissue samples derived from individuals with HIV-1 infection. These novel findings add significance to our previous findings that individuals with HIV-1-infection have low levels of GSH, GCLC and GSS in the red blood cells isolated from the peripheral blood [34].

We further analyzed the levels of MDA, a byproduct of lipid peroxidation due to free radical reaction in brain tissue samples from the three study groups. We observed a significant increase in the levels of free radicals in HIV-1-infected individuals compared to healthy and AD groups, but there was no significant difference between HIV-1 positive individuals with low and high CD4 sub groups (Fig. 5). Based on our findings, we conclude that an increased production of free radicals in HIV-1-infected individuals along with compromised levels of enzymes required for the synthesis of GSH along with the lack of GSR increase are mainly responsible for the significant decrease in the levels both total GSH and rGSH (Fig. 1A,B). The brain is more susceptible than any other organ in the body to oxidative damage and increased levels of free radicals along with diminished GSH will reduce the neuroprotection and may increase the risk for TBM. Many reports have linked redox imbalance as a pathogenic mechanism that contributes to AD [12], [23], [24]. However, we did not observe any increase in the levels of free radicals in the brain tissue samples derived from individuals with AD.

## Transparency document

Transparency document. Image 1
